# Real-World Evidence for Equality

**DOI:** 10.1089/heq.2020.0136

**Published:** 2021-10-06

**Authors:** C. Erwin Johnson, Yohance Omar Whiteside

**Affiliations:** Center for Observational and Real World Evidence, Merck & Co., Inc., Kenilworth, New Jersey, USA.

**Keywords:** real-world evidence, equality, racism, real-world data, health information technology, policy, anti-racism

## Abstract

As stakeholders in the transformative medical research ecosystem, real-world evidence researchers must conduct observational research with an awareness of racism. Advancements in understanding of the impact of racism on health outcomes, the abundance of health care data, and innovations in health information technology provide tools that create opportunities to conduct more focused research that illustrates how racism in health care deters the advancement of equity.

## Real-World Evidence Research to Address Inequity

Real-world evidence (RWE)^1^ researchers are stakeholders in the transformational medical research ecosystem. RWE research provides facts and evidence for decision and policy makers. The coronavirus pandemic and the cell phone documentation of police brutality that led to George Floyd's murder expose society's burdens of inequity, injustice, and racism. Health care in the United States is not immune to these burdens.^2^ The tradition of RWE research has identified racial disparities in obstetrics and gynecology,^3^ the challenges of minority participation in clinical trials,^4^ as well as racial and economic factors for COVID-19 disease outcomes.^5^ Building on experience, health care information technology, and a renewed recognition that it is important to measure racism, not socially assigned “race,”^6,7^ RWE researchers have the opportunity to generate trusted RWE that leads to evidence-based policies to undo racism that potentially address the more elusive problem: inequity. RWE research must now be conducted with an awareness of racism.

## The Real World That Needs Researching

Events of 2020 expose the legacy of racism and its impact on health.^8^ The inability to acknowledge and address the history and legacy of racism contributes to our contemporary systems of inequity in health care and limits the impact of medical science innovations, particularly for African Americans, Indigenous/Native peoples, and religious and immigrant groups (both past and present). The increased dialogue and commentary about the impact of race in medicine and health care are encouraging.^9^ Going forward, RWE research can address racism by acknowledging its presence in our real world, asking tough research questions that focus on dismantling racism, use 21st century digital health care/social data, and apply big data analytics tools/technologies to measure outcomes that move facts forward to provide evidence to policy makers.

## The Tough Research Questions

High quality research begins with posing important research questions. The tough research questions begin with acknowledgment that racism and racist medical research practices have sabotaged trust in medical research for certain groups of people. Step one in formulating tough research questions: RWE researchers must be educated about (and respect) the legacy and scars of the Tuskegee Study and its contemporary relevance in African American communities.^10^ Sadly, exploitative events with Henrietta Lacks justify some suspicions of medical research.^11^ Marginalized communities are aware of, and concerned about, biases in algorithms that limit access to potential life-saving resources.^12^ Researchers can consider questions that account for mistrust in participation in medical research.^13^ Step two: Build upon the work of veteran scholars of racism research in health care that provides guidance for evidence and research needs to measure racism in health care.^14^ RWE research has identified disparities and inequities. When researchers identify variations in outcomes based on socially assigned “race” categories, they must ask why multiple times.^15^ Step three: RWE researchers must acknowledge and account for skepticism about medical research in communities impacted by racism. Formulate research questions aligned with those who experience racism. Avoid speculation. Step four: RWE researchers must begin to understand and learn the power of antiracist thinking in health care as displayed in the United Kingdom^16^ and the United States.^17^ When the scientific research community understands the possibilities of antiracism, we broaden our curiosity about why humanity is being damaged by burdens that focus on socially assigned “race” groups.^18^

Empowered with awareness and new ways of thinking, RWE researchers can formulate better research questions that address racism. RWE researchers can respectfully engage and collaborate with victimized communities to partner for research. Through informed engagement, RWE researchers can actively listen to concerns and questions these communities have not only about the research process, but also how the experiences of racism and inequity impact their health. With this information, health care outcomes and research study endpoints that matter most to victims of racism can be identified and studied. Partnerships and collaborations with community members must be formed to socialize the RWE research process, ensuring transparency and equity in representation in RWE results. Without more direct engagement with communities victimized by racism, and research questions crafted from the perspective of those directly impacted by racism, current (and future) RWE research questions, though useful for regulatory decision making, will have limited impact on evidence-based policies that reduce inequities in health care. Despite these challenges, and missed opportunities of the past, RWE research must move forward to build new and better research capacity through transparency, collaboration, and engagement at more holistic levels with community and research organizations, human and behavior science disciplines, and include economic/financial implications of racism.^19^ Research capacity that prioritizes RWE studies that address racism and inequality can provide important peer-reviewed studies to help undo these historical and contemporary deterrents to better health outcomes.

## Real-World Data Opportunities and Challenges

The path to high-quality research goes through high-quality data. Real-world data (RWD)^20^ is the currency of transformative RWE and an important component of new and better research capacity that addresses racism and inequities. We have richer and more comprehensive RWD assets that can help answer our tougher RWE research questions. The RWE research community must demand data standards, data quality, and data aggregation practices that include all populations. Interoperability technologies can help create a digital health care data-supported RWE research agenda that links all relevant population data.^21^ Safe, secure, privacy-protected, and accurate data from Medicaid and Medicare sources must be more accessible to RWE researchers. Data vendors, policy makers, and technologists who organize RWD must understand that RWD must be representative and inclusive to account for all populations.^22^ Recent Office of the National Coordinator (ONC) information blocking rules improve all patients' access to their health care data.^23^ These rules can leverage safe and secure Sustainable Medical Applications and Reusable Technologies (SMART)^24^ to connect different health care data types (some controlled by patients) to gather additional sources of data to conduct RWE research that addresses racism and inequality. With linked, comprehensive, and representative RWD, big data analytics and technologies can analyze complex data sets^25^ that lead to new insights into the impact of inequity due racism in health care.

## Evidence for Antiracist and Equitable Policy

Just as tough research questions about why some “race” groups have different outcomes than others, high-quality antiracism research leverages 21st century RWD. The impact of RWE research, unlike randomized controlled trials research, is its use for patient- and population-level policy. Like our contemporary real world, RWD and RWE research are messy, difficult, and complicated. Despite these realities, RWE researchers must lean in with confidence that our skills, experiences, focus, better data, big data analytics capabilities, and our abilities to collaborate can collectively produce evidence that generates policies to dismantle systems of racism and inequity in health care. RWE research can build paths to policy for equality and help create an antiracist health care system.^26^

## Conclusion: 21st Century Direction

RWE researchers now have tools to generate RWE that can quantify, understand, and account for racism and its impact on health care outcomes, and generate data-driven evidence to influence important policies. The RWE research community is now armed with 21st century tools that include knowledge and awareness of the mistakes of our past, new ways to formulate antiracist research questions that focus on inequity, collaborations and partnerships with communities, science stakeholders (basic, medical, technology, and economic), digital health care, social data technology, and big data analytics. RWE researchers can feel emboldened to push forward into the challenge of conducting RWE research for equity. 21st century RWE research can leverage the best of our 21st century tools, and thinking, to generate RWE that leads to policies for a health care system that is antidehumanizing and more equitable for every human being.

## Abbreviations Used

RWD: real-world data
RWE: real-world evidence
